# The impact of zygote vitrification timing on pregnancy rate in frozen-thawed IVF/ICSI cycles

**DOI:** 10.3389/fcell.2023.1095069

**Published:** 2023-01-13

**Authors:** Sofia Makieva, Celine Stähli, Min Xie, Ana Velasco Gil, Maike Katja Sachs, Brigitte Leeners

**Affiliations:** ^1^ Department of Reproductive Endocrinology, University Hospital Zurich, Zurich, Switzerland; ^2^ Faculty of Medicine, University of Zurich, Zurich, Switzerland

**Keywords:** vitrification, bipronuclear, pregnancy, 2PN, zygote, ICSI, IVF, timing

## Abstract

**Introduction**: Cryopreservation of bipronuclear (2PN) stage zygotes is an integral part of IVF laboratory practice in countries with strict embryo culture legislation. Vitrification of zygotes is compatible with several strategies in infertility treatments holding a freeze-all indication and allows for effective workload management in settings with limited resources. Although it yields high survival rates and clinical outcomes, the ideal timing to commence vitrification of zygotes is elusive while it is empirically practiced in the window between 17 and 21 h post-insemination (hpi). We aimed to deduce the association between pregnancy rate and the time interval from insemination (IVF and ICSI) to vitrification to contribute to the standardization ofzygote cryopreservation.

**Methods**: A retrospective analysis of data on vitrification timings and pregnancy outcomes collected between 2011 and 2019 was performed. All included women received an embryo transfer after warming of vitrified zygotes at the 2PN stage.

**Results**: A total of 468 embryo transfers were included of which 182 (38.9%) resulted in pregnancy and 286 (61.1%) not. Vitrification was on average performed 18.74 ±0.63 hpi in the pregnant group and 18.62 ± 0.64 hpi in the non-pregnant group (OR 1.36, 95% CI 1.01; 1.83, *p* = 0.045). A multivariate analysis controlling for uterine pathologies, maternal age, AMH, the number of MII oocytes, previous history of pregnancy success, endometriosis, AFC, nicotine intake and male factor infertility showed no predictive value of vitrification timing on pregnancy rate. Three time intervals between insemination and vitrification were defined: 17:00 to 18:00 hpi (Group A), 18:01 to 19:00 hpi (Group B) and 19:01 to 21:00 hpi (Group C). Pregnancy occurred in 40/130 women (30.80%) in Group A, in 115/281 women (40.90%) in Group B and in 27/57 women (47.40%) in Group C. Univariate but not multivariate analysis showed a significantly higher pregnancy rate after the latest time interval between insemination and 2PN vitrification when compared to the earliest (Group C *vs*. A, OR 2.03, 95% CI 1.07; 3.84, *p* = 0.031).

**Discussion:** These findings encourage further investigation on the impact of vitrification timing on clinical outcomes and hold the potential to standardize cryopreservation of zygotes from IVF/ICSI cycles to eventually improve the quality of long-term ART outcomes.

## Introduction

Cryopreservation of bipronuclear (2PN) stage zygotes has an abiding role in IVF laboratory practice, independent of the ever-growing shift towards extended embryo culture (Al-Hasani et al., 2007). Indeed, 2PN stage cryopreservation inspires confidence in a plethora of clinical scenarios compatible with surplus embryo banking and freeze-all indication (Nikolettos and Al-Hasani, 2000; Vanderzwalmen et al., 2006). Advanced maternal age, low yield of zygotes, indication for cleavage stage transfer and previous history of no blastulation are some controversial justifications for elective incorporation of 2PN cryopreservation. To add to this controversy, some IVF laboratories cryopreserve at the 2PN stage for logistical reasons and workload management. Importantly, cryopreservation of zygotes circumvents national policies and religious reservations associated with extended embryo culture in various countries, whereby a set number of zygotes are permitted by law to remain in culture (Montag et al., 2006). For example in Germany only three zygotes are allowed to remain in culture assuming that the maximum number of embryos to be transferred is 3. Thus, law requires cryopreserving zygotes not intended to be transferred. The Swiss law also regulates embryo culture allowing a maximum of 12 zygotes to go forward. Consequently, bipronuclear stage cryopreservation is vital part of numerous clinics.

Bipronuclear stage cryopreservation exhibits above 90% survival rates after thawing due to the lack of spindle apparatus, unicellular form and the easy diagnosable survival by passage through syngamy and progression to first cleavage (Golakov et al., 2018). The only RCT that compared freeze-all clinical outcomes stemming from 2PN or blastocyst cryopreservation has found the two strategies equally effective (Shapiro et al., 2015). Importantly that study was done using the outdated slow freezing protocol. The same protocol was used from two older studies that directly compared outcomes from bipronuclear and cleavage stage freeze-thaw cycles finding bipronuclear stage freezing more effective (Troup et al., 1991; Senn et al., 2000). It is now clear that, for all developmental stages, vitrification is the best strategy for cryopreservation (Rienzi et al., 2017). However vitrification of zygotes in contrast to later stage embryos is time dependent as many important changes take place in the time interval between fertilization and first cleavage. Following sperm penetration, the oocyte completes the meiotic division, extrudes the second polar body and forms the 2PNs—male and female—that enter the G1 phase of the cell cycle. Timelapse studies have informed that 2PNs are evident at approximately six hpi with ICSI (Coticchio et al., 2018). Thereafter, the 2PNs enter the S phase to undergo DNA replication, which terminates when the zygote initiates the G2 phase approximately 18 hpi (Capmany et al., 1996). During the G2 phase, the zygote starts preparation for mitosis, including 2PN breakdown, which is observed approximately 23 hpi (Coticchio et al., 2018). It is crucial that zygotes are vitrified during the G2 phase and before 2PN breakdown (syngamy) to avoid the detrimental effects of freezing the embryo in a vulnerable state (Balakier et al., 1993). The G2 time interval of 18–23 h may seem ample, however, the aforementioned timings are approximate, relative to the insemination methodology and the intrinsic nature of some embryos to grow slower or faster. It is not unlikely that 2PNs change into syngamy soon after 18 hpi. The earliest PN breakdown has been noticed at 18 hpi and the latest between 30 and 31 hpi (Nagy et al., 1994; van Wissen et al., 1995; Capmany et al., 1996; Neuber et al., 2003). Interestingly, PN breakdown before 22 hpi was associated with higher clinical pregnancy compared to PN breakdown occurring between 22 and 25 hpi (Fancsovits et al., 2005). In contrast, in a more recent timelapse study, PN fading was shown to occur on average 24 hpi in zygotes resulting in live birth with no live births recorded when PN breakdown was occurring before 21 hpi (Azzarello et al., 2012). These variations in the critical checkpoints of the zygote cell cycle challenge the decision of when to cryopreserve. In this context, the first study to propose the best time for cryopreservation of zygotes was that of Wright and colleagues, who observed that zygotes slow-frozen after 23 hpi with IVF had 39% chance of pregnancy, which was significantly higher that the 13% observed before the aforementioned timepoint (Wright et al., 1990). Later, another study reported 24% pregnancy rate from 2PN stage zygotes cryopreserved between 16 and 23 hpi using slow freezing protocol (Miller and Goldberg, 1995). Others reported to cryopreserve zygotes approximately 20 hpi independent of IVF or ICSI (Macas et al., 1998; Damario et al., 1999) while 17 hpi was the time selected by a study vitrifying zygotes produced by conventional IVF (Dowling-Lacey et al., 2011).

Considering the variation in the timing of cryopreservation reported in the literature and the duration of the G2 phase, a reasonable strategy is thought to vitrify 2PN zygotes in the window between 17 and 21 hpi. However, the correlation of clinical outcomes with specific vitrification timings is currently unexplored. To the best of our knowledge, there are no studies examining the impact of the time interval between insemination and 2PN vitrification on chances for pregnancy. Herein we aimed to characterize the ideal vitrification timing for 2PN stage zygotes by exploring the impact of different time intervals between insemination and vitrification on clinical pregnancy rate.

## Materials and methods

### Study design

This is a retrospective cohort study investigating the association between the time interval between insemination of an oocyte and the vitrification of the 2PN stage zygote on the pregnancy rate in frozen-thawed transfer cycles. All study participants received a transfer of one or two embryos after thawing of 2PNs resulting from IVF or ICSI insemination between January 2011 and December 2019 at the University Hospital Zurich. Women were treated with a modified short, a long or an antagonist protocol. Healthcare professionals informed eligible women about the study, and asked them to participate. After a verbal agreement to evaluate relevant data, a written consent and confirming participation was provided by each participant. The manuscript was written according to the guidelines of Strengthening the Reporting of Observational studies in Epidemiology (STROBE) (von Elm et al., 2008).

### Inclusion and exclusion criteria

**FIGURE 1:**
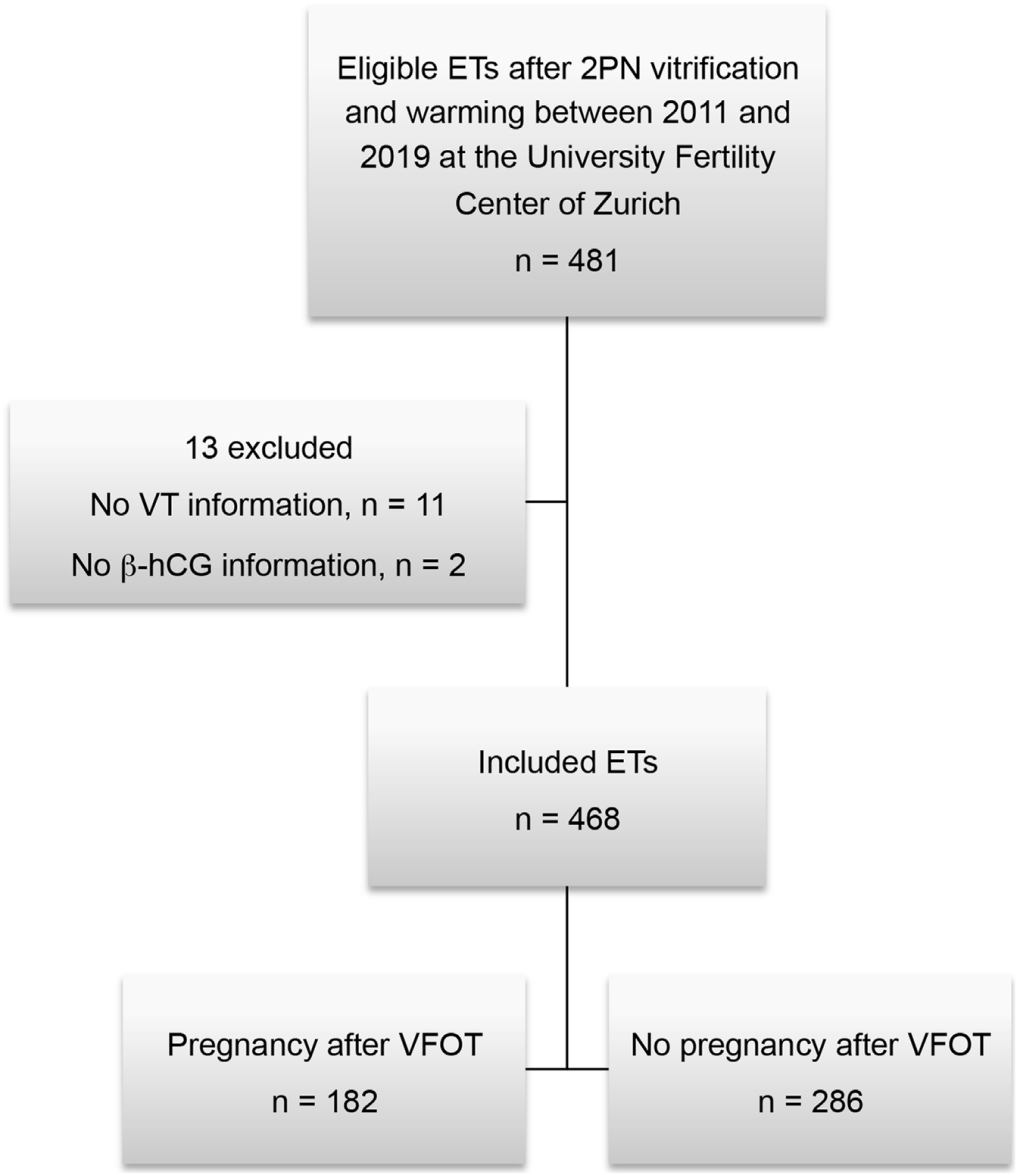
Overview of the study cohort. ET, embryo transfer; VT, vitrification time; VFOT, transfer after vitrified 2PNs.

Data were collected from women between 19 and 44 years of age (mean 35.1 ± 4.28 SD) who participated in an *in vitro* fertilization treatment. Women receiving vitrification of zygotes at 2PN stage with complete information on vitrification time and the endpoint pregnant or not pregnant were included in this evaluation. To allow independent data and exclusion of a “learning effect” from previous treatments as a possible confounder, only the first embryo transfer of each woman following cryopreservation in the study period was used for evaluation. Only vitrified 2PN stage zygotes but no cleavage stage or blastocysts were included. Of the 468 frozen-thawed transfer cycles included in the study, 182 resulted in a pregnancy and 286 did not (Figure 1).

### Controlled ovarian stimulation (COS) and endometrial preparation for transfer

Prior stimulation women received a gestagen (10 mg/d) for 12 days up to 4 weeks in the short or antagonist protocols and a GnRH-agonist (triptorelin, 0.1 mg/d) on cycle day 21 for long protocol COS. For COS, either short or long GnRH-agonist protocol or GnRH-antagonist protocol were used with either application of hMG or recombinant FSH. If at least three follicles with a diameter of ≥17 mm were observed during vaginal ultrasound, final oocyte maturation was induced with either 6500IE hCG (Ovitrelle, Merck, Switzerland) or with the addition of a GnRH-agonist accompanied by about 1600 IE hCG within the GnRH-antagonist protocol. Ultrasound guided oocyte retrieval was performed 35–37 h after administration of the hCG/GnRH-antagonist trigger. For artificial endometrial preparation, oral estrogen 6 mg/d followed by the addition of vaginal progesterone 1,000 mg/d prior to embryo transfer was administered. In natural cycle the transfer was performed on day 1 or day 2 after thawing, i.e., on day 2 (43–45 h) or day 3 (67–69 h) after insemination.

### Laboratory procedures

During ultrasound guided oocyte retrieval the transvaginal follicle aspiration needle (Vitrolife) was flushed between the two ovaries. The follicular fluid was collected in preheated round bottom 14 mL tubes held in a heating block calibrated at 37°C. The oocyte search was done under laminar flow using 60 mm flat bottom Petri dishes. All cumulus-oocyte complexes (COCs) were cultured in a humidified incubator with conditions of 37°C and 6% CO_2_ in fertilisation media (Global for Fertilisation, G-IVF Vitrolife) under oil (OVOIL, Vitrolife). Insemination was performed 4–6 h after oocyte retrieval by means of IVF or ICSI depending predominantly on the semen parameters. For ICSI, oocytes were denuded using hyaluronidase enzyme (80IU/mL) and following insemination incubated in Global Total or G1 Vitrolife media in microdroplet dish (Vitrolife) in MINC Benchtop incubator (COOK). For IVF, 10 × 10^6^ spermatozoa/ml were used to inseminate COCs distributed in wells of 4-well dishes in 700 ul of G-IVF or Global for Fertilisation media under oil. Sixteen to 19 h after insemination oocytes were inspected for the presence of two 2PN and two polar bodies. 2PN zygotes selected for immediate day 2/3 embryo transfer were left in culture. Surplus 2PN zygotes were subjected to vitrification for future thawing cycles. The presence of clear 2PNs was confirmed prior to the start of vitrification to avoid vitrifying zygotes in syngamy.

### Vitrification

The cryotop vitrification method was applied for all 2PN zygote cryopreservation. In this method, an open vitrification carrier, which contains a polypropylene strip accompanied by a protective cover, is used. By aspirating the excess solution, that is placed on the filmstrip, only a thin layer covering the cryopreserved cells ultimately remains. By using this minimal volume, the cooling rate is increased up to 2300°C/min and the warming rate up 4210°C/min (Kuwayama 2007). The cryotop method shows a high efficiency for the vitrification, causing only minimal cryodamage. The Kitazato vitrification (VT601) and thawing (VT602) solutions and the Cryotop^®^ Oocyte/Embryo Vitrification Device Open System were used for this study. No more than two zygotes at the 2PN stage were loaded per cryotop and thawed for embryo transfer. 2PN zygotes were cryopreserved between 17 and 21 hpi.

### Definitions

Oocytes in metaphase II (MII) were considered as mature and fertilizable. MII oocytes after IVF was a sum of oocytes that resulted in fertilisation and the oocytes that did not result in fertilisation in the presence of clear polar body. The following indications for fertility treatment were included in our analysis: primary or secondary sterility, endometriosis (with different rASRM stages), uterine and tubular pathologies, oocyte maturation problems, male and idiopathic infertility. A previous transfer was considered successful if it resulted in pregnancy. Based on the time interval between insemination and vitrification, vitrified zygote transfers were divided into a Group A with a time interval between 17:00 and 18:00 h, a Group B with a time interval of 18:01–19:00 h and a Group C with a time interval between 19:01 and 21:00 hpi. The vitrification time was defined as the mean time from start to the end of insemination procedure. The primary outcome was the pregnancy rate (biochemical and clinical). Biochemical pregnancies were defined by hCG level greater than 10 mlU/ml 14 days after embryo transfer (negative test <0, 3 mIU/mL). Clinical pregnancy was diagnosed with an intrauterine gestational sac confirmed by transvaginal ultrasound. The body mass index (BMI = kg/m^2^) was classified according to the standards of the World Health Organization. i.e., overweight was defined as a BMI >24.9.

### Sample size

Basedon comparable published studies, we estimated that the proportion of pregnancy rate for the shortest time group (17:00 to 18:00 hpi) should be approximately 25%–30% (Al-Hasani et al., 2007). Cohen’s h served as the measure for effect size between the two proportions (Cohen J., 1998). A standard power and sample size computation was used to calculate the number of participants needed to detect different effect sizes between the group with the earliest and the latest time interval between insemination and vitrification with adequate statistical power (at least 80%) and a significance level of alpha = .05 in a two-sided hypothesis test. Differences of 10% in the pregnancy rate between two groups would be detected at 80% statistical power and a similar significance level with each sample including 329 embryo transfers. Differences of 15% between the two pregnancy rates would be detectable with comparable statistical power with a cohort size of *n* = 152 and detection of a 20% difference would require 89 embryo transfers in each group. The study was able to enroll 468 vitrified 2PN zygote transfers: 130 in the group with the earliest time interval between insemination and vitrification and 57 in latest time interval. Thus, the retrospective cohort does not allow for statistically powerful comparisons to detect differences less than 20% between group proportions. The present analysis therefore should be interpreted on the aforementioned basis.

### Data analysis

Statistical tests were carried out using the statistical program SPSS, Version 26: An IBM software platform for advanced statistical analysis. Outcomes are presented as absolute numbers (n), percentages (%), 95% confidence intervals and as mean±standard deviation. Observations are quantitatively summarized in descriptive statistics. Differences in ordinal variables across the two groups and differences between the means were tested by the non-parametric Wilcoxon Mann-Whitney test, categorical variables with the Chi-squared test. Fisher Exact tests were used when comparing 2 × 2 contingency tables or the group to be examined had a smaller number than five. When appropriate, a two-tailed test was used. To analyze the relationship between the outcome variable (incidence of pregnancy) and independent variables, univariate and multivariate logistic regressions were performed. Associations are shown by odds ratio and adjusted for confounders.

First, a univariate analysis was performed with all the covariates. All those significant at a significance level of alpha = .05 in the univariate analysis were included in the model for logistic multivariate analysis. Any hypothesis test was considered significant at the alpha = .05 significance level.

### Ethical approval

The study was approved by the local ethics committee of the Canton of Zurich (BASEC Nr. 2018-00796).

## Results

### Study population characteristics

Data from 468 embryo transfers after vitrification and thawing of 2PN stage zygotes were analyzed. Altogether, 182 embryo transfers resulted in pregnancy and 286 embryo transfers did not (Figure 1). Thus, the overall pregnancy rate was 35.6%. Of the 182 transfers with a positive pregnancy result, 139 led to live births. The socio-demographic characteristics were comprehensively compared between women who achieved a pregnancy and those who did not. Maternal age (*p* = .004) and smoking at the time of the fertility treatment (*p* = .049) differed significantly between the two groups (Table 1). Women who achieved pregnancy presented less uterine pathologies and endometriosis but they had significantly more frequent indication for male factor infertility (Table 2). Moreover the pregnant women in our cohort had higher Antral Follicle Count (AFC) and Anti-Müllerian hormone (AMH) compared to women who did not get pregnant after frozen-thawed cycle (Table 3). Finally, the two groups differed significantly in the amount of mature oocytes (MII) available for treatment and previous history of a positive pregnancy (Table 4).

**TABLE 1:** Socio-epidemiologic characteristics of cohort.

	Pregnancy after ET after 2PN vitrification-warming *n* = 182 (38.9%)	No pregnancy after ET after 2PN vitrification-warming *n* = 286 (61.1%)	*p*-value
Age in years	*n* = 139	*n* = 246	
Mean in years (±σ)	34.31 (±4.32)	35.63 (±4.18)	.004
BMI (kg/m^2^)	*n* = 139	*n* = 246	
Mean in kg/m^2^ (±σ)	22.47 (±3.22)	22.90 (±3.54)	.341
Overweight (Yes)	28 (20.10%)	61 (24.80%)	.298
Nationality	*n* = 134	*n* = 243	
Swiss, Lichtenstein	73 (54.5%)	137 (56.4%)	.736
German	17 (12.7%)	21 (8.6%)	
Other	44 (32.8%)	85 (35.0%)	
Smoking	*n* = 137	*n* = 245	
Never smoked/Former smoker	128 (93.4%)	213 (86.9%)	.049
Current smoker	9 (6.6%)	32 (13.1%)	

ET, embryo transfer.

Wilcoxon-Mann-Whitney test = independent *t*-test for age and BMI (ordinal variables), Chi-squared-test for nationality and smoking (categorical variables).

Overweight BMI >24.9 kg/m^2^.

**TABLE 2:** Overview of indications for fertility treatment.

		Pregnancy after ET after 2PN vitrification-warming *n* = 182 (38.9%)	No pregnancy after ET after 2PN vitrification-warming *n* = 286 (61.1%)	*p*-value
Sterility		*n* = 136	*n* = 243	
Primary		90 (66.2%)	144 (59.3%)	.184
Secondary		46 (33.8%)	99 (40.7%)	
Endometriosis		*n* = 137	*n* = 244	
	Yes	19 (13.9%)	55 (22.5%)	.040
		*n* = 16	*n* = 52	
rASRM stage I		2 (12.5%)	18 (34.6%)	.099
rASRM stage II		4 (25.0%)	13 (25.0%)	
rASRM stage III		7 (43.75%)	13 (25.0%)	
rASRM stage IV		3 (18.75%)	8 (15.4%)	
Uterine pathologies		*n* = 137	*n* = 244	
	Yes	21 (15.3%)	77 (31.6%)	.001
Tubular pathologies		*n* = 137	*n* = 244	
	Yes	22 (16.1%)	46 (18.9%)	.494
Disorder follicle maturation^a^		*n* = 182	*n* = 286	
	Yes	17 (9.3%)	23 (8.0%)	.624
Male Cause		*n* = 137	*n* = 243	
	Yes	69 (50.4%)	97 (39.9%)	.049
Idiopathic		*n* = 137	*n* = 244	
	Yes	6 (4.4%)	11 (4.5%)	.953

ET, embryo transfer.

Chi-squared-test for all variables (categorical variables).

^a^ Anovulation and Oligo-/Amenorrhea.

**TABLE 3:** Overview of basic conditions for fertility treatment.

	Pregnancy after ET after 2PN vitrification-warming *n* = 182 (38.9%)	No pregnancy after ET after 2PN vitrification-warming *n* = 286 (61.1%)	*p*-value
Fertilization method	*n* = 115	*n* = 159	
ICSI	104 (90.4%)	143 (89.9%)	.209
IVF	10 (8.7%)	10 (6.3%)	
Both	1 (.9%)	6 (3.8%)	
AFC	*n* = 100	*n* = 170	
Mean in # follicles (±σ)	14.46 (±8.09)	12.96 (±7.48)	.042
Period cycle	*n* = 121	*n* = 227	
Minimum; Mean in # days (±σ)	29.14 (±7.61)	28.17 (±5.34)	.090
Maximum; Mean in # days (±σ)	32.60 (±12.46)	30.40 (±8.67)	.110
Basal hormone levels			
Basic E2	*n* = 118	*n* = 191	
Mean in pg/L (±σ)	65.16 (±49.91)	65.55 (±55.85)	.725
FSH	*n* = 129	*n* = 223	
Mean in IE/L (±σ)	6.63 (±2.00)	7.03 (±2.66)	.074
Basic Progesterone	*n* = 118	*n* = 191	
Mean in ng/L (±σ)	1.32 (±.67)	1.23 (±.76)	.095
AMH	*n* = 113	*n* = 204	
Mean in pmol/L (±σ)	23.85 (±24.69)	17.05 (±15.75)	.005
Androstenedione	*n* = 26	*n* = 29	
Mean (±σ)	8.69 (±4.61)	6.77 (±5.30)	.074
DHEAS	*n* = 28	*n* = 31	
Mean (±σ)	5.24 (±2.35)	4.67 (±2.51)	.284

ET, embryo transfer.

Wilcoxon-Mann-Whitney test for ordinal variables, Chi-squared-test for categorical variables.

**TABLE 4:** Results of fertility treatment.

		Pregnancy after ET after 2PN vitrification-warming *n* = 182 (38.9%)	No pregnancy after ET after 2PN vitrification-warming *n* = 286 (61.1%)	*p*-value
MII oocytes		*n* = 117	*n* = 188	
		12.21 (±6.14)	10.55 (±5.43)	.012
Previous successful fresh transfer^a^		*n* = 45	*n* = 98	
	Yes	43 (95.6%)	81 (82.7%)	.035
Number of previous miscarriages		*n* = 137	*n* = 234	
	0	112 (81.8%)	191 (81.6%)	.921
	1	18 (13.1%)	28 (12.0%)	
	2	3 (2.2%)	5 (2.1%)	
	>2	4 (2.9%)	10 (4.3%)	

ET, embryo transfer.

Wilcoxon-Mann-Whitney test for ordinal variables, Chi-squared-test for categorical variables.

MII, metaphase II oocytes.

^a^ Resulted in a pregnancy.

### Vitrification between 19 and 21 hpi is beneficial

The average time from insemination to vitrification was significantly higher in women who achieved pregnancy compared to those who did not; 18.74 ± .63 hpi vs. 18.62 ± .64 hpi, *p* = .038 (Table 5). To further explore this initial observation, we performed a univariate analysis to assess the association between vitrification timing as a continuous variable and the pregnancy rate and found this to be significant with OR 1.36, 95%-CI 1.01; 1.83 and *p* = .045. However, statistical significance was lost in multivariate analysis taking into consideration the differences in the socio-demographic characteristics of the groups (Table 6).

**TABLE 5:** Overview of hourly averages.

	Pregnancy after ET after 2PN vitrification-warming *n* = 182 (38.9%)	No pregnancy after ET after 2PN vitrification-warming *n* = 286 (61.1%)	*p*-value
Vitrification time^a^	*n* = 182	*n* = 286	
Mean in hours (±σ)	18.74 (±.63)	18.62 (±.64)	.038
Group A (*n* = 130)	*n* = 40	*n* = 90	
Mean in hours (±σ)	17.94 (±.17)	17.91 (±.26)	.816
Group B (*n* = 281)	*n* = 115	*n* = 166	
Mean in hours (±σ)	18.77 (±.25)	18.78 (±.25)	.838
Group C (*n* = 57)	*n* = 27	*n* = 30	
Mean in hours (±σ)	19.77 (±.31)	19.85 (±.40)	.567

ET, embryo transfer.

Wilcoxon-Mann-Whitney test for difference between the means.

^a^ Continuous variable.

Group A: Vitrification time 17:00—18:00 hpi, B: Vitrification time 18:01—19:00 hpi, C: Vitrification time 19:01—21:00 hpi.

**TABLE 6:** Uni- and multivariate analysis with vitrification time as continuous variable.

Predictor	Crude OR (95% CI)	Crude *p*-values	Adjusted OR (95% CI)^a^	Adjusted *p*-values
Pregnancy rate				
Vitrification time^b^	1.36 (1.01; 1.83)	**.045**	1.38 (.90; 2.13)	.142
Uterine pathologies	.39 (.23; .67)	**.001**	.55 (.25; 1.20)	.132
Age	.93 (.89; .98)	**.004**	.95 (.89; 1.02)	.144
AMH	1.02 (1.01; 1.03)	**.006**	1.01 (.99; 1.03)	.287
MII oocytes	1.05 (1.01; 1.10)	**.015**	1.01 (.96; 1.07)	.626
Previous successful fresh transfer^c^	4.51 (1.00; 20.45)	.051	—	—
Endometriosis	.55 (.31; .98)	**.042**	.92 (.40; 2.10)	.839
AFC	1.03 (1.00; 1.07)	.051	—	—
Nicotine	.47 (.22; 1.01)	.054	—	—
Male cause	1.53 (1.00; 2.33)	**.049**	1.12 (.59; 2.13)	.734

^a^ Adjusted for vitrification time (continuous variable), uterine pathologies, age, AMH, MII, endometriosis, and male cause.

^b^ Continuous variable.

MII, metaphase II oocytes.

^c^ Resulted in a pregnancy.

These are raw *p* values. Crude *p* < 0.05 is considered significant. The adjusted *p* value is after correction for factors that can influence the result. Adjusted *p* < 0.05 is considered significant.

**FIGURE 2:**
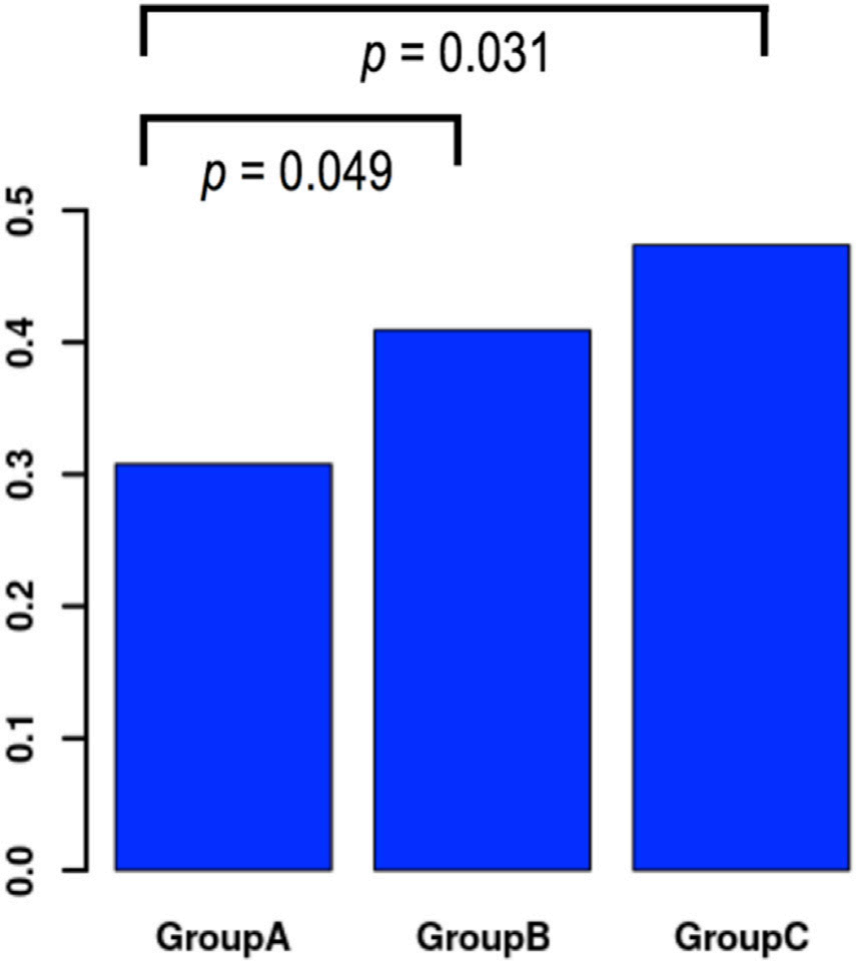
Distribution of pregnancy results in temporal groups and comparison of success rates across groups in univariate analysis. Group A: Vitrification time 17:00–18:00 hpi, Group B: Vitrification time 18:01–19:00 hpi, Group C: Vitrification time 19:01–21:00 hpi.

To examine whether a time interval could be set as a threshold before commencing vitrification, we initially performed a subgroup analysis based on three time intervals from insemination to vitrification (Table 5). In Group A vitrification commenced between 17:00 and 18:00 hpi with an average time 17.94 ± .17 hpi in the pregnant group. In Group B vitrification commenced between 18:01 to 19:00 hpi with an average time 18.77 ± .25 hpi in the pregnant group. In Group C vitrification commenced between 19:01 to 21:00 hpi with an average time 19.77 ± .25 hpi in the pregnant group. The average times between pregnant and not pregnant women were similar in each one of the three subgroups. The pregnancy rates among the three groups were 30.80% for Group A, 40.90% for Group B and 47.40% for Group C (Table 7). A univariate analysis showed that the chance of pregnancy was significantly associated with the three groups (OR 1.45, *p* = .018), but the significance vanished after multivariate analysis (Table 8). Comparison of the latest vs. the earliest time interval showed an OR of 2.03 (*p* = .031), for the longest vs. the middle group an OR of 1.30 (*p* = .370) and for the middle vs. the earliest time interval an OR of 1.56 (*p* = .049) in univariate analysis (Table 9; Figure 2). In the multivariate analysis these observations were not confirmed.

**TABLE 7:** Distribution of pregnancy results after different time intervals between insemination and vitrification.

	Pregnancy after ET after 2PN vitrification-warming *n* = 182 (38.9%)	No pregnancy after ET after 2PN vitrification-warming *n* = 286 (61.1%)
Group A (*n* = 130)	*n* = 40 (30.80%)	*n* = 90 (69.20%)
Group B (*n* = 281)	*n* = 115 (40.90%)	*n* = 166 (59.10%)
Group C (*n* = 57)	*n* = 27 (47.40%)	*n* = 30 (52.60%)

ET, embryo transfer.

Group A: Vitrification time 17:00–18:00 hpi, B: Vitrification time 18:01–19:00 hpi, C: Vitrification time 19:01–21:00 hpi.

**TABLE 8:** Uni- and multivariate analysis with vitrification time divided into three groups.

Predictor	Crude OR (95% CI)	Crude *p*-values	Adjusted OR (95% CI)^a^	Adjusted *p*-values
Pregnancy rate				
Vitrification time^b^	1.45 (1.07; 1.97)	**.018**	1.38 (.88; 2.15)	.157
Uterine pathologies	.39 (.23; .67)	**.001**	.56 (.26; 1.22)	.145
Age	.93 (.89; .98)	**.004**	.95 (.89; 1.02)	.169
AMH	1.02 (1.01; 1.03)	**.006**	1.01 (.99; 1.03)	.308
MII oocytes	1.05 (1.01; 1.10)	**.015**	1.02 (.96; 1.07)	.613
Previous successful fresh transfer^c^	4.51 (1.00; 20.45)	.051	—	—
Endometriosis	.55 (.31; .98)	**.042**	.93 (.41; 2.12)	.862
AFC	1.03 (1.00; 1.07)	.051	—	—
Nicotine	.47 (.22; 1.01)	.054	—	—
Male cause	1.53 (1.00; 2.33)	**.049**	1.14 (.60; 2.17)	.691

^a^ Adjusted for vitrification time (groups), uterine pathologies, age, AMH, MII, endometriosis, and male cause.

^b^ Divided into three groups.

MII, metaphase II.

^c^ Resulted in a pregnancy.

These are raw *p* values. Crude *p* < 0.05 is considered significant. The adjusted *p* value is after correction for factors that can influence the result. Adjusted *p* < 0.05 is considered significant.

**TABLE 9:** Comparison of the success rates across temporal groups.

Predictor	OR (95% CI)	Crude *p*-value^a^	Adjusted OR (95% CI)^b^	Adjusted *p*-values	*p*-value^c^
Pregnancy rate					
Group C vs. A	2.03 (1.07; 3.84)	**.031**	2.03 (.80; 5.16)	.135	**.033**
Group C vs. B	1.30 (.96; 1.98)	.370	1.63 (.75; 3.87)	.256	.381
Group B vs. A	1.56 (1.00; 2.43)	**.049**	1.24 (.66; 2.33)	.497	.050
Comparison across A, B, C	—	—	—	—	.054^d^

^a^ *p*-values of regression (comparison of the groups).

^b^ Adjusted for vitrification time (groups), uterine pathologies, age, AMH, MII, endometriosis, and male cause.

^c^ Two-tailed Fisher exact test.

^d^ Chi-squared test, two degrees of freedom.

Group A: Vitrification time 17:00–18:00 hpi, B: Vitrification time 18:01–19:00 hpi, C: Vitrification time 19:01–21:00 hpi.

## Discussion

Cryopreservation of zygotes has a conserved role in IVF laboratory practice due to its flexibility spanning circumvention of laws surrounding embryo culture and opportunity to expand the repertoire of treatment strategies with a freeze-all indication. Compared to oocytes, 2PNs are more efficient in absorbing cryoprotectant, releasing water and exhibit a better tolerance to osmotic pressure during thawing, thus poised to be less susceptible to damage (Chen et al., 2003; Diedrich et al., 2020). What is more, cleavage stage embryos perform worse at vitrification in comparison to blastocysts, suggesting that banking of 2PNs for future cleavage stage transfers would be advantageous (Zeng et al., 2018). Zygotes should be strictly vitrified in the G2 phase of the cycle before PN breakdown but the ideal precise time has never been defined and clinical outcomes associated with vitrification timing have never been investigated.

We present an unprecedented study on the impact of time interval from insemination to vitrification on pregnancy rates in freeze-thaw IVF/ICSI cycles. We show that the chance of pregnancy was not influenced by a precise timing within the 17 to 21 hpi window recommended for 2PN stage vitrification, when all relevant factors influencing pregnancy were considered. However, it is important to emphasize that the odds of getting pregnant were more than twice as high in embryo transfers where vitrification was realized after 19:01 hpi and before 21:00 hpi compared to earlier than 18:00 hpi in a univariate analysis.

We believe that the discordance between uni- and multivariate analyses is attributed to our sample size and retrospective nature of the study. Our power analysis showed that a larger cohort would be beneficial to reliably investigate the association between the time intervals from insemination to vitrification with pregnancy chance. Indeed the complex multivariate analysis, with some of the confounding factors showing rather low prevalence, would preferentially be realized within a larger dataset. Credibility for this notion comes from the thorough assessment of the confounders in our study groups—pregnant and non-pregnant. It is remarkable that some very well-known influencing factors for pregnancy such as maternal age, AMH concentration, uterine pathology and the number of mature oocytes (Surrey et al., 2005; Chen et al., 2018; Coccia et al., 2020) failed to show a statistically significant influence on pregnancy chance in the multivariate model, despite being significant in the univariate analysis. History of a previous successful embryo transfer increased chances for a positive pregnancy test in our cohort, which is additionally in agreement with previous studies (Bushaqer et al., 2020). Male factor infertility was complementary found to be significantly associated with pregnancy chances in our univariate analysis, which could be successfully corrected by ICSI. In Group C (19:01 to 21:00 hpi) only 57 transfers were realized, i.e., differences <20% between groups were not adequately powered and it was unlikely that the effect would reach a 20% difference. Hence, the small sample size and the uneven distribution of cases in the different timing groups are the main limiting factors in the pursue of a reliable multifactorial analysis. Unfortunately, our sample size did not allow controlling for IVF vs. ICSI insemination methods. Subgroup analysis based on insemination method would be based on the hypothesis that IVF and ICSI morphokinetic parameters, for example, the timing of PNBD, are different. Indeed some studies suggest marginal time differences between the two insemination methods, which would be explained by the fact that in IVF sperm does not have immediate access to the oocyte cytoplasm as it does with ICSI (Bodri et al., 2015). However, there are also studies arguing that PNBD timing is independent of the insemination method (Karlikaya et al., 2022). This could be explained by the fact that even 30 s of gamete co-incubation for conventional IVF are enough to produce fertilization and clinical outcomes comparable to the longer traditional gamete co-incubation condition (Bungum et al., 2006). It is tempting to hypothesize that sperm penetration in IVF is faster than assumed and that the difference in time of insemination for IVF and ICSI is marginal (De Munck et al., 2022). What is more, pooling IVF and ICSI data and suggesting one time point for zygote freezing that accommodates both methods, would significantly facilitate simplification of routine laboratory practice. One of the strengths of our study includes the high quality of the data due to the fertilization treatments being all performed at one university fertility center, employing same standards for all patients. Data quality was further increased by including only the first thawing cycle in a woman during the study period to reduce the impact of any “learning effect.”

Regardless of the result of the multivariate analysis, and due to the overall concordance that we detected in the confounder univariate analysis with the literature, we have confidence in the interpretation of our findings that prompt refraining from vitrifying zygotes too early in the G2 phase but instead encourage allowing at least 19 h from insemination to elapse. This observation is in line with the physiological changes occurring in the zygote as it transitions from the G1 to the M phase of the cell cycle. The exact timing for the initiation and end of the G2 phase cannot be defined due to zygote-to-zygote variation and insemination method. However, timelapse studies have informed that 2PNs first appear on average six hpi and disappear 23 hpi with ICSI while the fading of 2PNs signifies the entry of the zygote into the M phase of the cycle (Coticchio et al., 2018). A wide variety of cryopreservation timings have been reported in the literature within the window from 16 to 23 hpi with IVF or ICSI following the slow freezing or vitrification procedure (Miller and Goldberg, 1995; Macas et al., 1998; Damario et al., 1999; Dowling-Lacey et al., 2011). Balakier et al. (1993) suggested that the S phase of the zygote cycle, characterized by active DNA synthesis in the 2PNs, is initiated by approximately 9–10 hpi and is completed 3–5 h later. During this phase, zygotes are susceptible to damage and should never be exposed to cryopreservation, proposing that the ideal time for the procedure would be 20 to 22 hpi when the majority of zygotes are found in G2 phase before PN breakdown and chromosome condensation. A later study reported the S phase to commence between 8 and 14 hpi and last 2–4 h (Capmany et al., 1996). According to these studies the S phase of the zygote could be expected to be completed anywhere between 10 and 18 hpi. The earliest PN breakdown has been reported at 18 hpi and the latest between 30 and 31 hpi (Nagy et al., 1994; van Wissen et al., 1995; Capmany et al., 1996; Neuber et al., 2003). This great degree of variation adds a layer of complexity when it comes to the optimization of the ideal timing for vitrification, which empirically has been practiced between 17 and 21 hpi, soon after fertilization check and before PN breakdown. Our result suggests that the chance of pregnancy is significantly lower when zygotes at 2PN stage are vitrified before 18 hpi when compared to after 19 hpi. This difference might be explained by the possibility that some zygotes in the Group A (17:00-18:00 hpi) cohort have been vitrified while still at the end of the S phase. Unfortunately it is not possible to capture the transition from S to G2 phase in zygotes with timelapse technology (Gallego et al., 2019) although such technology has been developed and used in other cell types (Sakaue-Sawano et al., 2008). Until such timelapse option is available to use for human embryos to confirm our hypothesis, we suggest that vitrification past 19 hpi would be the safest strategy to avoid cryopreserving the cell during a fragile stage. Our suggested timing is even more important in light of a recent scientific breakthrough showing that 2PN zygotes initiate transcription before 18 hpi (Asami et al., 2022). Specifically, Asami et al. showed that embryonic gene activation (EGA) is in fact triggered by fertilization and is not inactive until the embryo advances to the 8-cell stage as it was previously thought. They also reported that the very first genes transcribed at the zygote are associated with genes involved in cancer pathways. Thus, impaired EGA, for example due to vitrification, could possibly have detrimental impact on offspring health. Indeed vitrification has been shown to alter RNA transcription in oocytes (Huo et al., 2021) and accumulating evidence is emerging of an link between frozen-thawed embryo transfer with childhood cancer (Sargisian et al., 2022). Therefore, an ideal timing to perform vitrification of zygotes may be defined as the one that is the least invasive when it comes to interfering with the delicate and unique to zygote molecular machineries. It would be interesting to examine the impact of vitrification timing on neonatal outcomes from transfers of thawed zygotes to further unravel the aforementioned speculation.

In conclusion, we observed that waiting until 19 hpi before commencing vitrification at the 2PN zygote stage could improve the chance of pregnancy. Our result contributes to the elucidation of the ideal vitrification timing that could help to avoid jeopardizing the developmental potential of the transferred embryo. We encourage the community to confirm our findings using larger datasets to allow evaluation of the relevance of vitrification time in the context of the relevant confounders. In light of recent evidence linking childhood cancer to frozen-thawed embryo transfer; optimizing vitrification beyond the endpoint of post-warming survival and pregnancy chance should become top priority. Our findings call for biosafety awareness directed towards those performing routinely zygote vitrification during the empirically set timeframe between 17 and 21 hpi with a reminder that ART success is defined as the achievement of a pregnancy resulting in a healthy offspring.

## Data Availability

The raw data supporting the conclusion of this article will be made available by the authors, without undue reservation.
